# Durability Analysis of a Self-Expanding Transcatheter Aortic Valve Through 30 Years of Accelerated Wear Testing

**DOI:** 10.1016/j.shj.2026.101084

**Published:** 2026-06-25

**Authors:** Guilherme Attizzani, Marco Frazzetto, Ole De Backer, Danny Dvir, Vanessa Garlepp, Tim Kelley, Kendra J. Grubb, Gilbert H.L. Tang

**Affiliations:** a Division of Cardiology, University Hospitals of Cleveland, Case Western Reserve University, Cleveland, Ohio, USA; b Osaka University, Osaka, Japan; c The Heart Center, Rigshospitalet, Copenhagen, Denmark; d Institute of Clinical Medicine, Copenhagen University, Copenhagen, Denmark; e Interventional Cardiology Department, Shaare Zedek Medical Center, Hebrew University of Jerusalem, Jerusalem, Israel; f Medtronic, Mounds View, Minnesota, USA; g Department of Cardiovascular Surgery, Mount Sinai Health System, New York, New York, USA

**Keywords:** Heart valve prosthesis, Prosthesis durability, Transcatheter aortic valve replacement

## Abstract

• First reported 30-year in vitro accelerated wear testing data for a THV.
• All Evolut PRO + valves met ISO hydrodynamic criteria through 20 years of testing.
• At least one valve per size maintained performance through 30 years of testing.
• Failures manifested in a consistent pattern, suggesting a shared mechanism.
• Testing is ongoing until all valves fail.

As transcatheter aortic valve replacement expands to younger and lower-risk patients, prosthesis durability is central to lifetime management. Despite rapid technological advances, durability beyond 10 years remains unknown.^1^ This gap is particularly relevant for patients who may outlive the expected lifespan of current transcatheter heart valves (THVs). In the absence of long-term clinical durability data, in vitro bench testing provides mechanistic and comparative insights into THV structural and functional longevity. Accordingly, we evaluated the long-term (through 30 equivalent years) in vitro durability of nominally deployed Medtronic Evolut PRO+ valves using accelerated wear testing.

Evolut PRO+ valves were tested according to International Organization for Standardization (ISO) 5840:2021 guidelines.^2^ Valves were fixtured at the midpoint of the indicated annulus range (nominal interference) and tested under nominal deployment (circular conditions) at a target differential pressure of 100 mm Hg and a 16-Hz cyclic frequency through 1.2 billion cycles (equivalent to 30 years). Three valves per size (23, 26, 29, and 34 mm) were evaluated.

All Evolut valves underwent inspection, environmental conditioning with distribution simulation, and simulated delivery system use (loading and 2 recapture cycles) before testing. At 50-million-cycle intervals, valves underwent visual inspection and hydrodynamic assessment, including measurements of transvalvular regurgitant fraction (RF) and effective orifice area. Valves that failed to meet minimum ISO hydrodynamic performance requirements at any time point were deemed failures and excluded from further analysis from that point onward.

All 12 Evolut valves met ISO performance requirements through 20 years (800 million cycles) of accelerated wear testing (Figure 1b and c). Seven of 12 Evolut valves successfully completed testing through 30 years (1.2 billion cycles). The remaining 5 valves exceeded maximum RF thresholds at prespecified time points: 1 26-mm valve at 20.6 years, 2 29-mm valves (1 at 23.8 and 1 at 30.0 years), and 2 34-mm valves (1 at 28.8 and 1 at 30.0 years). These valves were excluded from subsequent analyses. All valves met ISO effective orifice area requirements through 1.2 billion cycles.

**Figure 1:**
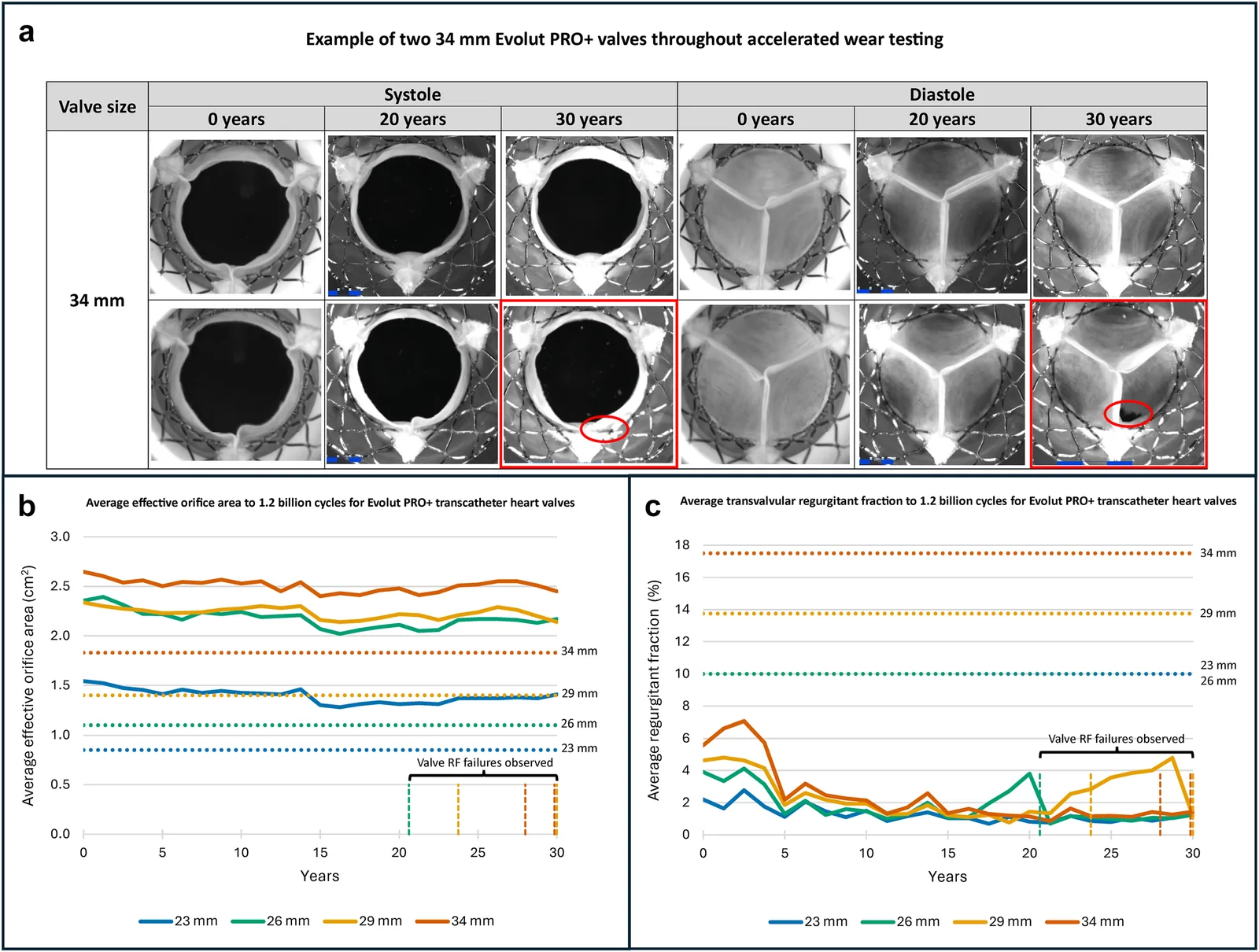
**Accelerated durability testing of Evolut PRO+ transcatheter heart valves.** Three Evolut PRO+ valves per size (23 mm, 26 mm, 29 mm, and 34 mm) underwent AWT. (**a**) Representative images of 2 34-mm valves throughout AWT, demonstrating a valve meeting all acceptance criteria through 30 years (top) and a valve failing at approximately 22 years (bottom) with red circles highlighting leaflet damage. Average EOA (**b**) and average transvalvular RF (**c**) through 1.2 billion cycles (equivalent to 30 years) were assessed via hydrodynamic testing at 50-million-cycle intervals. Horizontal dashed lines indicate the maximum allowable RF under ISO 5840:2021 guidelines for each valve size. Vertical dashed lines denote the time point at which a valve was removed from subsequent analysis because the RF value exceeded ISO criteria. Valves exceeding ISO criteria were excluded from mean calculations at the point of failure and thereafter. Abbreviations: AWT, accelerated wear testing; EOA, effective orifice area; ISO, International Organization for Standardization; RF, regurgitant fraction.

Among Evolut valves completing the 30-year time point, visual inspection revealed minor wear typical of tissue valves in accelerated durability assessments, with no impact on valve function (Figure 1a). In the 5 valves that failed for exceeding RF thresholds, leaflet tears were observed at the leaflet tip in 1 valve (26 mm) and at the suture margin in 4 valves (2 29 mm and 2 34 mm). Failures manifested in a consistent pattern, suggesting a shared mechanism and location of wear under accelerated loading. Testing is ongoing until all valves fail, which will further define the temporal onset of degradation and refine mechanistic understanding.

This in vitro study cannot replicate in vivo factors such as calcification, thrombosis, and pannus formation. Due to aggressive cycling rates and controlled conditions, wear patterns may be exaggerated or may differ from those observed in vivo. Testing was performed under circular annulus conditions at nominal deployment, which does not always reflect clinical practice where treatment of noncircular anatomies is common. Additionally, although 3 valves per size group satisfies the ISO 5840:2021 testing standard, this cohort size constrains the ability to perform statistical analyses within the test groups.

In conclusion, all 12 Evolut valves across all sizes met ISO performance requirements through the equivalent of 20 years (800 million cycles), confirming the durability of the supraannular, self-expanding platform. Most Evolut valves, including at least 1 valve of each Evolut size, met all performance criteria through 30 years, representing the longest published durability data for any THV platform to date.^3^^,^^4^ These findings provide incremental evidence to inform lifetime management as transcatheter aortic valve replacement expands to younger populations.

## Ethics Statement

This research adhered to the relevant ethical guidelines.

## Funding

Bench testing was funded by 10.13039/100004374Medtronic.

## Disclosure Statement

G. Attizzani has served as a consultant, advisory board member, and research grant recipient for Medtronic and has served as a consultant for Dasi Simulations, FEOPS, Elixir, and Abbott Vascular. O. De Backer has received research grants and served as a consultant for Abbott, Boston Scientific, Medtronic, and MicroPort. D. Dvir has served as a consultant for Medtronic, Edwards Lifesciences, Abbott, and Pi-Cardia. V. Garlepp is a Medtronic employee. K.J. Grubb is a Medtronic employee. T. Kelley is a Medtronic employee. G.H.L. Tang has received speaker honoraria and served as a physician proctor, consultant, advisory board member, TAVR publications committee member, RESTORE study steering committee member, APOLLO trial screening committee member, and IMPACT MR steering committee member for Medtronic; has received speaker honoraria and served as a physician proctor, consultant, advisory board member, and TRILUMINATE trial anatomic eligibility and publications committee member for Abbott Structural Heart; has served as an advisory board member for Boston Scientific and JenaValve; has served as a consultant and physician screening committee member for Shockwave Medical; and has served as a consultant for Anteris, Philips, Edwards Lifesciences, Pi-Cardia, Peija Medical, and Shenqi Medical Technology. The other authors have no conflicts to declare.

## References

[1] Thyregod H.G.H., Jorgensen T.H., Ihlemann N. (2024). Transcatheter or surgical aortic valve implantation: 10-year outcomes of the NOTION trial. Eur Heart J.

[2] International Organization for Standardization (2021).

[3] Sathananthan J., Hensey M., Landes U. (2020). Long-term durability of transcatheter heart valves: insights from bench testing to 25 years. JACC Cardiovasc Interv.

[4] Sathananthan J., Landes U., Flaction L. (2021). Long-term durability of the next-generation acurate neo 2 transcatheter heart valve: insights from bench testing to 25 years. JACC Cardiovasc Interv.

